# Supplementary material for: Investigations of Histomonosis-Favouring Conditions: A Hypotheses-Generating Case-Series-Study
Source: Animals (Basel). 2023 Apr 26;13(9):1472. doi: 10.3390/ani13091472 (PMC10177171; doi:10.3390/ani13091472)
Supplement: Supplementary file 1 [file animals-13-01472-s001.zip › P3_Supplementary Materials.pdf]

Table S1. Description and management of the farm and flock.

Variable	Variable category	Case-farms (n = 31)	
		n	%
Farm description and -management			
Type of the farm	Conventional rearing	2	6.45
	Conventional fattening	12	38.71
	Conventional rearing and fattening	15	48.39
	Alternative rearing and fattening	1	3.23
	Conventional rearing and breeder husbandry	1	3.23
Availability of an outdoor climate area	Yes	0	0.00
	No	31	100.00
Availability of free-range	Yes, during the outbreak	1	3.23
	No	30	96.77
Multiple outbreaks on the farm	No	13	41.94
	Yes, 1 to 2 months ago	1	3.23
	Yes, 5 to 6 months ago	3	9.68
	Yes, 6 to 12 months ago	6	19.35
	Yes, more than 12 months ago	8	25.81
Heating system of the turkey house	Biogas heat	8	25.81
	Gas (open)	9	29.03
	Gas (closed)	10	32.26
	Warm water	4	12.90
Flock description and -management			
Sex of the flock	Male	21	67.74
	Female	2	6.45
	Male and female	8	25.81
Management of placement	First shared houses, later splitting in different houses	10	32.26
	Directly placement in different houses	14	45.16
	Directly placement in different parts of a house, later splitting in different houses	7	22.58
Movement of a flock 10 days before and during an outbreak	Yes	10	32.26
	No	21	67.74
Environmental conditions			
Unmaintained green area around the turkey house	Yes	1	3.23
	No	30	96.77
Well-maintained green area around the turkey house	Yes	28	90.32
	No	3	9.68
Trees and bushes around the turkey house	Yes	21	67.74
	No	10	32.26
Vegetation-free ground around the turkey house	Yes	3	9.68
	No	28	90.32

Variable	Variable category	Case-farms (n = 31)	
		n	%
Concrete and/or asphalt around the turkey house	Yes	26	83.87
	No	5	16.13
Gravel around the turkey house	Yes	3	9.68
	No	28	90.32
Paving stones around the turkey house	Yes	15	48.39
	No	16	51.61
Increased precipitation 10 days before and during an outbreak	Yes	3	9.68
	No	28	90.32
Persistent drought 10 days before and during an outbreak	Yes	12	38.71
	No	19	61.29
Increased outside temperature 10 days before and during an outbreak	Yes	4	12.90
	No	27	87.10
Decreased outside temperature 10 days before and during an outbreak	Yes	0	0.00
	No	31	100.00
Strong wind 10 days before and during an outbreak	Yes	5	16.13
	No	26	83.87
Breakdown of ventilation 10 days before and during an outbreak	Yes	0	0.00
	No	31	100.00
High humidity in the turkey house 10 days before and during an outbreak	Yes	0	0.00
	No	31	100.00
Feed- and water management			
Breakdown of feeding- and/or drinking lines 10 days before and during an outbreak	Yes	0	0.00
	No	31	100.00
Leakage of the waterline up to 10 days before outbreak	Yes	1	3.23
	No	30	96.77
Change of feed before outbreak	1 week before	11	35.48
	2 weeks before	14	45.16
	3 weeks before	4	12.90
	More than 3 weeks before	2	6.45
Coccidiostatics in the feed up to 10 days before and during outbreak	Yes	23	74.19
	No	8	25.81
Sensorial abnormalities in the feed	Yes	0	0.00
	No	31	100.00

Table S2a. General biosecurity measures.

Variable	Variable category	Case-farms (n = 31)	
		n	%
Turkey house conditions			
Plastic on the ground or on the lower walls in the turkey house	Yes, sleek	4	12.90
	No	27	87.10
Metal on the ground or on the lower walls in the turkey house	Yes	0	0.00
	No	31	100.00
Wood on the ground or on the lower walls in the turkey house	Yes	0	0.00
	No	31	100.00
Concrete on the ground or on the lower walls in the turkey house	Yes, sleek	8	25.81
	Yes, sleek with cracks	12	38.71
	Yes, sleek and rough with/without cracks	3	9.68
	Yes, rough with cracks	8	25.80
Vectors			
Observation of earthworms under disinfection basins in the hygiene lock	Yes	0	0.00
	No	26	83.87
	No disinfection installation	5	16.13
Insect control in the turkey house	Yes	7	22.58
	No	24	77.42
Observation of insects in the turkey house (flies, earthworms, beetles, snails, others)	Yes, during heating	1	3.23
	Yes, in the first fattening part	1	3.23
	Yes, in the second fattening part	1	3.23
	Yes, during the whole fatting time	1	3.23
	Yes, after the turkeys were moved to slaughter	10	32.26
	No	17	54.84
Machines driving into the turkey house before the outbreak	Yes, 1 day before	9	29.03
	Yes, 2-5 days before	5	16.13
	Yes, more than 6 days before	2	6.45
	No	15	48.39
Number of animal keepers	One person per farm	26	83.87
	Two persons per farm	5	16.13
Changes of animal keepers up to 10 days before an outbreak	Yes	0	0.00
	No	31	100.00
Hygiene lock and equipment			
Location of hygiene lock	Farm entry	1	3.23
	House entry	26	83.87
	Farm entry and house entry	4	12.90
Washbasin in the hygiene lock	Yes	30	96.77
	No	1	3.23
Soap in the hygiene lock	Yes	27	87.10
	No	4	12.90

Variable	Variable category	Case-farms (n = 31)	
		**n**	**%**
Disinfectant in the hygiene lock	Yes	10	32.26
	No	21	67.74
Shower in the hygiene lock	Yes	3	9.68
	No	28	90.32
Turkey house-specific overall in the hygiene lock	Yes	13	41.94
	No	18	58.06
Turkey house-specific footwear in the hygiene lock	Yes	30	96.77
	No	1	3.23
One-way footwear in the hygiene lock	Yes	8	25.81
	No	23	74.19
One-way gloves in the hygiene lock	Yes	4	12.90
	No	27	87.10
One-way overall in the hygiene lock	Yes	7	22.58
	No	24	77.42
One-way hairnet in the hygiene lock	Yes	2	6.45
	No	29	93.55
Disinfection installation for footwear in the hygiene lock	Yes	26	83.87
	No	5	16.13
Cleaning and disinfection			
Cleaning frequency of the clothes	Daily	2	6.45
	Weekly	18	58.06
	Monthly	7	22.58
	Less frequent than monthly	3	9.68
	Between placements	1	3.23
Cleaning frequency of the turkey house-specific footwear	Daily	4	12.90
	Weekly	4	12.90
	Monthly	1	3.23
	Between placements	21	67.74
	No data	1	3.23
Cleaning frequency and fill up of disinfection installation for footwear	Daily	1	3.23
	Several times a week	6	19.35
	Weekly	9	29.03
	Less frequent than weekly	10	32.26
	No disinfection installation	5	16.13
Cleaning frequency of the hygiene lock	Several times a week	6	19.35
	Weekly	11	35.48
	Less frequent than weekly	5	16.13
	Between placements	9	29.03
Disinfection frequency of the hygiene lock	Several times a week	3	9.68
	Weekly	3	9.68
	Less frequent than weekly	3	9.68
	Between placements	22	70.97

Variable	Variable category	Case-farms (n = 31)	
		**n**	**%**
Waste water management	Catch- or seepage pit	23	74.19
	Slurry pit	7	22.58
	Distribution on green areas around the turkey house	1	3.23
Regular cleaning of the cadaver storage place	Yes	28	90.32
	No	3	9.68
Regular disinfection of the cadaver storage place	Yes	27	87.10
	No	4	12.90
Cleaning frequency of the feed silo	More frequently than annually	2	6.45
	Annually	3	9.68
	Every 2-5 years	13	41.94
	Less frequently than every 5 years	7	22.58
	Never	6	19.35
Enrichment materials			
Usage of hay as enrichment material	Yes	1	3.23
	No	30	96.77
Usage of straw as enrichment material	Yes	6	19.35
	No	25	80.65
Usage of popcorn as enrichment material	Yes	0	0.00
	No	31	100.00
Usage of fresh plants as enrichment material	Yes	0	0.00
	No	31	100.00
Usage of plastic as enrichment material	Yes	13	41.94
	No	18	58.06
Usage of wood as enrichment material	Yes	1	3.23
	No	30	96.77
Usage of metal as enrichment material	Yes	1	3.23
	No	30	96.77
Usage of pecking stones as enrichment material	Yes	21	67.74
	No	10	32.26
Usage of spelt pellets as enrichment material	Yes	2	6.45
	No	29	93.55
Usage of grit as enrichment material	Yes	1	3.23
	No	30	96.77
Usage of elevated level as enrichment material	Yes	1	3.23
	No	30	96.77
Usage of CDs as enrichment material	Yes	1	3.23
	No	30	96.77
Usage of egg cardboard as enrichment material	Yes	1	3.23
	No	30	96.77

Variable	Variable category	Case-farms (n = 31)	
		n	%
Usage of rubber as enrichment material	Yes	1	3.23
	No	30	96.77
Re-use of enrichment material	Yes	12	38.71
	No	19	61.29

Table S2b. General biosecurity measures.

Variable	Case-farms (n = 31)						
	**median**	**standard deviation**	**variance coefficient**	**minimum**	**25%-quantile**	**75%-quantile**	**maximum**
Turkey house conditions							
Number of turkey houses per farm	3.00	1.69	45.23	1.00	3.00	5.00	8.00
Time between outbreaks in different houses per farm (days)	1.50	8.67	199.67	0.00	0.00	3.00	28.00
Distance to turkey farms, which were affected at the same time (km)	3.50	3.01	60.17	2.00	2.50	7.00	10.00
Cleaning and disinfection							
Average duration of service period in general (days)	12.50	7.47	46.91	8.50	10.50	21.00	42.00

n = number

km = kilometres

Table S3a. Health management, incidence and therapy of diseases.

Variable	Variable category	Case-farms (n = 31)	
		**n**	**%**
	General veterinary care		
Veterinary visit up to 10 days before an outbreak	Yes	26	83.87
	No	5	16.13
	General vaccination program		
Flock was vaccinated against Haemorrhagic Enteritis before outbreak	Yes	20	64.52
	No	11	35.48
Flock was vaccinated against Turkey Rhinotracheitis before outbreak	Yes	23	74.19
	No	8	25.81
Flock was vaccinated against Newcastle Disease before outbreak	Yes	30	96.77
	No	1	3.23
Flock was vaccinated against turkey house-specific pathogens before outbreak	Yes	10	32.26
	No	21	67.74
Flock was vaccinated against avian encephalomyelitis before outbreak	Yes	1	3.23
	No	30	96.77
Flock was vaccinated against Reovirus before outbreak	Yes	2	6.45
	No	29	93.55
Flock was vaccinated against *Salmonella* spp. before outbreak	Yes	1	3.23
	No	30	96.77
Flock was vaccinated against *Escherichia coli* before outbreak	Yes	4	12.90
	No	27	87.10
Continuation of the vaccination programme during outbreak	Yes	12	38.71
	No	19	61.29
	Pre-existing diseases		
Pre-burden of the flock in general	Yes	23	74.19
	No	8	25.81
Pre-burden of the flock by omphalitis	Yes	1	3.23
	No	30	96.77
Pre-burden of the flock by respiratory disease	Yes	2	6.45
	No	29	93.55
Pre-burden of the flock by gastrointestinal disease	Yes	22	70.97
	No	9	29.03
Pre-burden of the flock by disease of the locomotion system	Yes	0	0.00
	No	31	100.00
Pre-burden of the flock by systemic disease	Yes	3	9.68
	No	28	90.32
Pre-burden of the flock by pecking	Yes	0	0.00
	No	31	100.00

Variable	Variable category	Case-farms (n = 31)	
		n	%
Pre-burden of the flock by non-starting problem	Yes	1	3.23
	No	30	96.77
Pre-existing pathogens			
Pre-burden of the flock by *Escherichia coli*	Yes	19	61.29
	No	12	38.71
Pre-burden of the flock by *Eimeria* spp.	Yes	14	45.16
	No	17	54.84
Pre-burden of the flock by *Clostridium* spp.	Yes	8	25.81
	No	23	74.19
Pre-burden of the flock by *Heterakis gallinarum*	Yes	0	0.00
	No	31	100.00
Diagnostic of pre-existing diseases and of histomonosis			
Usage of necropsy as diagnostic tool	Yes	31	100.00
	No	0	0.00
Usage of bacteriological examination as diagnostic tool	Yes	5	16.13
	No	26	83.87
Usage of parasitological examination as diagnostic tool	Yes	2	6.45
	No	29	93.55
Usage of PCR as diagnostic tool	Yes	28	90.32
	No	3	9.68
Usage of epidemiological investigations as diagnostic tool	Yes	6	19.35
	No	25	80.65
Usage of feed examination as diagnostic tool	Yes	0	0.00
	No	31	100.00
Detection of histomonosis			
Positive detection of histomonosis by necropsy	Yes	31	100.00
	No	0	0.00
Positive detection of histomonosis by parasitological examination	Yes	0	0.00
	No	31	100.00
Positive detection of histomonosis by PCR	Yes	27	87.10
	No	4	12.90
Treatment against pre-existing diseases			
Pretreatment of the flock in general	Yes	29	93.55
	No	2	6.45
Pretreatment of the flock by antibiotics	Yes	24	77.42
	No	7	22.58
Pretreatment of the flock by antiparasitics	Yes	25	80.65
	No	6	19.35

Variable	Variable category	Case-farms (n = 31)	
		n	%
Pretreatment of the flock by acids in the drinking water	Yes	10	32.26
	No	21	67.74
Pretreatment of the flock by other methods	Yes	1	3.23
	No	30	96.77
	Treatment against histomonosis		
Treatment against histomonosis	Yes, since first clinical hints	10	32.26
	Yes, since first diagnostic hints	17	54.83
	Yes, since elevated mortality rate	3	9.68
	No	1	3.23
Co-treatment of non-affected turkey houses	Yes	13	41.94
	No	18	58.06
Treatment with Paromomycin	Yes	27	87.10
	No	4	12.90
Treatment with analgesic drugs	Yes	1	3.23
	No	30	96.77
Treatment with copper sulphate	Yes	0	0.00
	No	31	100.00
Treatment with oregano	Yes	14	45.16
	No	17	54.84
Treatment by emergency killing	Yes	10	32.26
	No	21	67.74
Treatment by emergency slaughter	Yes	0	0.00
	No	31	100.00
Treatment with vitamins and electrolytes	Yes	3	9.68
	No	28	90.32
Treatment with pre- and probiotics	Yes	0	0.00
	No	31	100.00
Treatment with salts	Yes	0	0.00
	No	31	100.00
Treatment by sobering	Yes	0	0.00
	No	31	100.00
Treatment success	Yes, very fast	5	16.13
	Yes, only hesitantly	8	25.81
	No	17	54.84
	Untreated flock	1	3.23

Table S3b. Health management, incidence and therapy of diseases.

Variable	Case-farms (n = 31)						
	**median**	**standard deviation**	**variance coefficient**	**minimum**	**25%-quantile**	**75%-quantile**	**maximum**
General vaccination program							
Time between the last vaccination and the beginning of outbreak (days)	14.00	14.63	86.08	0.00	7.00	24.00	78.00
Clinical onset and mortality of histomonosis							
Age of turkeys at outbreak (days of life)	47.00	23.56	46.33	19.00	33.00	62.00	110.00
Mortality rate (%)	25.00	30.10	81.00	4.00	10.00	54.00	100.00
Period of increased mortality (days of life)	21.00	25.81	95.70	0.00	5.00	37.00	98.00
Time of first clinical symptoms (days of life)	46.00	24.89	48.32	19.00	34.00	58.00	113.00
Detection of histomonosis							
Time of first pathological hints (days of life)	46.00	25.24	48.99	20.00	32.00	58.00	114.00

n= number

Table S4. Outbreak management.

**Variable**	**Variable category**	**Case-farms (n = 31)**	
		**n**	**%**
	Biosecurity measures		
Usage of turkey house-specific clothes in case of histomonosis	Yes	30	96.77
	No	1	3.23
Usage of turkey house-specific equipment in case of histomonosis	Yes	19	61.29
	No	12	38.71
Repassing the hygiene lock after disposal of cadaver	Yes	28	90.32
	No	3	9.68
	Flock management		
Number of animal checks per day	Twice	22	70.97
	Three times	5	16.13
	More than three times	4	12.90
Animal check frequency compared between weekdays and weekend or holidays	Equal frequently	29	93.55
	Less frequently	2	6.45
Animal check frequency compared between healthy and sick flocks	Equal frequently	11	35.48
	More frequently	20	64.52
Separation of ill turkeys in the same turkey house in case of histomonosis	Yes	5	16.13
	No	26	83.87
Separation of ill turkeys in another turkey house in case of histomonosis	Yes	0	0.00
	No	31	100.00
Increased killing in case of histomonosis	Yes	29	93.55
	No	2	6.45
Climate management of the turkey house during the outbreak	Constant temperature	23	74.19
	Increased temperature	8	25.81
Activation of the humidity system during the outbreak	Yes	7	22.58
	No	17	54.84
	No humidity system in the turkey house	7	22.58
	Cadaver management		
Removal of cadaver in case of histomonosis	Yes	31	100.00
	No	0	0.00
Usage of hands for cadaver removal	Yes	31	100.00
	No	0	0.00
Usage of a wheelbarrow for cadaver removal	Yes	12	38.71
	No	19	61.29
Usage of a wheel loader for cadaver removal	Yes	5	16.13
	No	26	83.87
Cadaver transport through the turkey house door	Yes	21	67.74
	No	10	32.26

Variable	Variable category	Case-farms (n = 31)	
		n	%
Cadaver transport through the hygiene lock	Yes	12	38.71
	No	19	61.29
Cadaver transport through emergency- and maintenance doors	Yes	9	29.03
	No	22	70.97
Cooled storage of cadaver	Yes	29	93.55
	No	2	6.45
Closed storage of cadaver	Yes	31	100.00
	No	0	0.00
Cadaver storage on plan-fortified ground	Yes	31	100.00
	No	0	0.00
Dung management			
Application of the dung on agricultural land	Yes	6	19.35
	No	25	80.65
Introduction of the dung into a biogas system	Yes	23	74.19
	No	8	25.81
Incineration of the dung	Yes	0	0.00
	No	31	100.00
Previous disinfection of the dung	Yes	2	6.45
	No	29	93.55

Table S5a. Coincidental findings.

Variable	Variable category	Case-farms (n = 31)	
		**n**	**%**
Increased observation of earthworms around the turkey house 10 days before and during an outbreak	Yes	4	12.90
	No	27	87.10
Waterlogging in the turkey house10 days before and during an outbreak	Yes	4	12.90
	No	27	87.10
Observation of wild birds in the turkey house before outbreak	Yes	3	9.68
	No	28	90.32
Activities on the fields around the turkey house 10 days before and during an outbreak	Yes	5	16.13
	No	26	83.87
Usage of fresh straw before outbreak	Yes	5	16.13
	No	26	83.87
Vaccination team visit up to 10 days before an outbreak	Yes	4	12.90
	No	27	87.10

Table S5b. Coincidental findings.

**Variable**	**Case-farms (n = 31)**						
	**median**	**standard deviation**	**variance coefficient**	**minimum**	**25%-quantile**	**75%-quantile**	**maximum**
Turkey house conditions							
Distance to turkey farms, which were affected at the same time (km)	3.50	3.01	60.17	2.00	2.50	7.00	10.00

n= number

km= kilometres
